# Recent progress in biodiversity research on the Xylariales and their secondary metabolism

**DOI:** 10.1038/s41429-020-00376-0

**Published:** 2020-10-23

**Authors:** Kevin Becker, Marc Stadler

**Affiliations:** 1 Department Microbial Drugs, Helmholtz Centre for Infection Research GmbH, Inhoffenstraße 7, 38124 Braunschweig, Germany; 2 German Centre for Infection Research Association (DZIF), partner site Hannover-Braunschweig, Inhoffenstraße 7, 38124 Braunschweig, Germany

**Keywords:** Metabolomics, Fungi

## Abstract

The families Xylariaceae and Hypoxylaceae (Xylariales, Ascomycota) represent one of the most prolific lineages of secondary metabolite producers. Like many other fungal taxa, they exhibit their highest diversity in the tropics. The stromata as well as the mycelial cultures of these fungi (the latter of which are frequently being isolated as endophytes of seed plants) have given rise to the discovery of many unprecedented secondary metabolites. Some of those served as lead compounds for development of pharmaceuticals and agrochemicals. Recently, the endophytic Xylariales have also come in the focus of biological control, since some of their species show strong antagonistic effects against fungal and other pathogens. New compounds, including volatiles as well as nonvolatiles, are steadily being discovered from these ascomycetes, and polythetic taxonomy now allows for elucidation of the life cycle of the endophytes for the first time. Moreover, recently high-quality genome sequences of some strains have become available, which facilitates phylogenomic studies as well as the elucidation of the biosynthetic gene clusters (BGC) as a starting point for synthetic biotechnology approaches. In this review, we summarize recent findings, focusing on the publications of the past 3 years.

## Introduction

Recent studies relying on bioinformatics, molecular ecology, and phylogenetics have revealed a very high species diversity in the fungal kingdom, and according to some estimates, there are several million undescribed species [1]. Recent studies using high-throughput and third generation sequencing techniques are now able to depict the diversity very accurately and often hitherto unknown phylogenetic lineages of fungi are detected [2, 3]. It often has been postulated that these unknown fungal organisms may constitute a great resource for new enzymes, drugs, agrochemicals, and other useful natural molecules, but by far not all groups of fungi have a diverse secondary metabolism. This has only been proven for certain taxonomic entities like the order Xylariales, from which a large number of unique carbon skeletons have already been discovered during the course of classical natural product screening programmes.

**Fig. 1:**
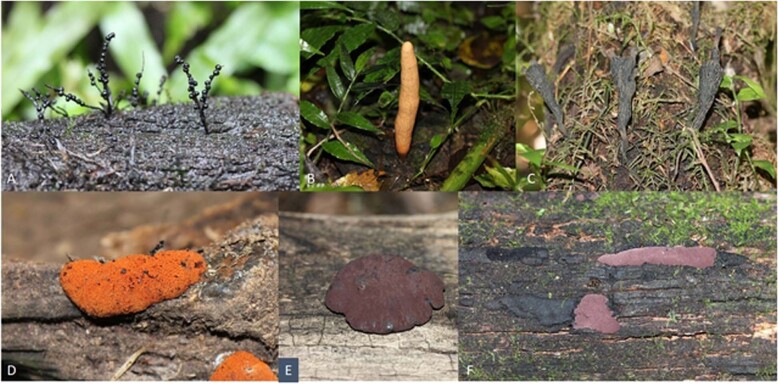
Stromata of some tropical and subtropical species of Xylariales. **a** *Xylaria melanura*. **b** *X. telfairii*. **c** *X. grammica*. **d** *Hypoxylon haematostroma*. **e** *Pyrenopolyporus hunteri*. **f** *H. griseobrunneum*. Images were kindly provided by Esteban B. Sir

The current paper follows up on the review by Helaly et al. [4], where the state-of-the-art on secondary metabolite discovery and various correlations to biodiversity research had been described for this fungal order, taking the most important papers that were published until 2017 into account. Only 3 years later, a lot of new information has accumulated and it is already time for an update. The present paper is roughly divided in three parts, the first two of which are dealing with interesting new compounds from the two large families within Xylariales, Xylariaceae, and Hypoxylaceae (for illustrations of representative species see Fig. 1), and the third part covers recent taxonomic, chemoecological, and phylogenomic studies of these fungi.

## Novel secondary metabolites from the Xylariaceae *sensu stricto* (Fig. 2)

After recent taxonomic revisions, in which other families like the Hypoxylaceae have been excluded, and only counting the genera that have been studied by molecular phylogenetic methods or examination of the asexual stages, the Xylariaceae *sensu strictu* presently comprise 33 genera and over 1000 (and up to 1230) species, of which more than 50% belong to the genus *Xylaria* [5, 6]. The number of accepted *Xylaria* taxa vary from 570 to 670 in these recent overviews, depending on whether synonyms and variations are counted. The genus *Xylaria* has never been subjected to a world monograph using modern, polythetic methodology. From the outcome of such work in other genera of the Xylariales, it is possible that *Xylaria* actually comprises several thousands of species of which the majority remains to be recognized and formally described [7].

**Fig. 2:**
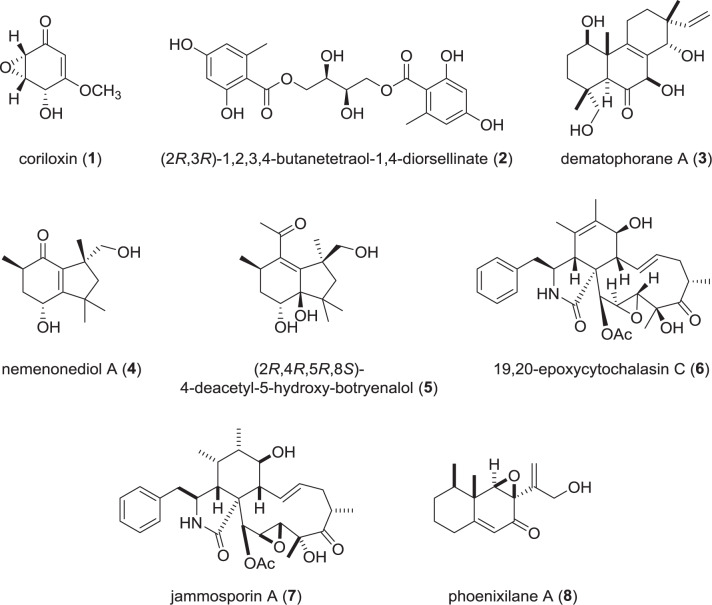
Secondary metabolites recently described from the genera *Amphirosellinia*, *Ascotricha*, *Dematophora*, *Nemania*, and *Stromatoneurospora*

For this reason, it is not surprising that most of the new metabolites reported from the family were derived from *Xylaria* species, as highlighted in a comprehensive review some years ago [4], for which we provide an update from the past years. It is important to note that most of the metabolites of *Xylaria* reported over the past decade have actually been isolated from endophytic strains.

Besides *Xylaria*, only for some genera of the Xylariaceae novel secondary metabolites have been described in the past 3 years. Those included *Nemania*, *Rosellinia*, and *Dematophora*, the latter of which has recently been resurrected and emended [8]. Most other genera, even larger ones like *Hypocopra* and *Kretzschmaria*, are nearly untapped concerning their secondary metabolites. This may be the case because they have rarely been collected or because they are not easy to cultivate.

Most of the compounds described today are still discovered by the classical method of axenic cultivation, either in submerged culture or on solid media, and successive extraction. However, other approaches like biotransformation, co-cultivation, epigenetic modification, and heterologous expression are encountered occasionally and will be discussed herein as well.

Subsequently, the genera for which novel secondary metabolites have been described in the past 3 years will be dealt with, and secondary metabolites by their affiliation to different compound classes. Some papers published prior to 2018 will be included herein for the sake of integrity in concurrence with the latest review [4], which covers most of the literature on secondary metabolites from Xylariales up to the end of 2017. We preferentially treat compounds that were not already reported previously [4, 9, 10].

The genus *Amphirosellinia* was not studied for secondary metabolites until recently, when the first natural product, coriloxin (**1**), was discovered from cultures of *Amphirosellinia* *nigrospora* [11]. Coriloxin represents a known cyclohexenone derivative [12] and was found to have weak antimicrobial activities. However, the crude extract of the fungus showed stronger bioactivities than the purified compound against phytopathogenic bacteria like *Ralstonia solanacearum* and plant-pathogenic fungi like *Magnaporthe oryzae*, suggesting either the presence of additional bioactive compounds in the extract and/or synergistic effects of **1** with other components. Those results render the producing organism *A. nigrospora* an interesting target for further research on its biocontrol capabilities against crop diseases.

*Ascotricha* was already described to produce orsellinic acid-glucosides in 2017, where short-time cultivation of a sea mud-derived *Ascotricha* sp. in submerged media led to isolation of two glucosides comprised of orsellinic acid and d-threitol, *e.g*. (2*R*,3*R*)-1,2,3,4-butanetetraol-1,4-diorsellinate (**2**) [13]. For **2**, even a synthesis was described shortly after [14]. No biological activities were reported for those compounds from *Ascotricha*; however, they represent the first natural products from a genus that is otherwise still untapped.

Species of *Dematophora* were up to recently included in the genus *Rosellinia*, but were segregated due to differing asexual morphs and a well-defined clade in phylogenetic analyses [8]. The same paper also described two novel bioactive isopimarene diterpenoids from *D. bunodes*, (previously known as *R. bunodes*) named dematophoranes A (**3**) and B. The compounds showed weak antibacterial activity against *Bacillus subtilis* and *Staphylococcus aureus* and weak cytotoxic activities against mouse fibroblasts (cell line L929) and cervix carcinoma cells (KB 3.1). Other purified but known isopimarenes like myrocin B and the glucoside hymatoxin K were already reported from other species of Xylariales and Hypocreales. In addition, the presence of additional compounds with MS data that could not be matched to the entries in the available natural product databases was observed, making *Dematophora* spp. a worthwhile genus to examine more thoroughly.

*Nemania* is one of the largest genera in the Xylariaceae and some of its species are relatively well-studied. Recently, an endopyhtic isolate named *Nemania bipapillata* isolated from a red alga was described to produce botryane-type sesquiterpenoids [15]. This class of compounds had so far not been described from this genus, but from species of *Hypoxylon* and *Daldinia*, both belonging to the Hypoxylaceae [16, 17]. Among the compounds isolated from *N. bipapillata*, a *nor*-sesquiterpene named nemenonediol A (**4**) showed the most potent inhibition of human acetylcholinesterase (27.7% inhibition at 100 µM) in comparison to the reference galanthamine (90% at 100 µM) [15]. Furthermore, the sesquiterpene (2*R*,4*R*,5*R*,8*S*)-4-deacetyl-5-hydroxy-botryenalol (**5**) showed the strongest effect among the isolated botryanes against human butyrylcholinesterase (7.3% inhibition at 100 µM vs. 82% in the galanthamine control).

Cytochalasans are a class of hybrid polyketide-non-ribosomal peptide (PKS-NRPS) natural products of fungal origin, of which many representatives are known for their activity against the eukaryotic actin skeleton [18]. Cytochalasans are distributed over many genera within Xylariales, including the large families Xylariaceae and Hypoxylaceae. In 2019, the known 19,20-epoxycytochalasin C (**6**) and D, as well as 18-deoxy-19,20-epoxycytochalasin C were described from an endophytic *Nemania* sp. [19]. These three cytochalasans were found to have antiplasmodial and phytotoxic activities, but also high toxicity against mammalian cells. However, it was also shown elsewhere that the cytotoxicity against the actin skeleton is not irreversible for several cytochalasans like (**6**) [18]. In addition, recently discovered biological effects like the inhibition of biofilm formation in the human pathogenic bacterium *Staphylococcus aureus* must be due to other mechanisms of action because bacteria do not possess an actin cytoskeleton [20]. This also concerned the recently isolated sacchalasins from stromata of *Daldinia sacchari*, an endemic species of South Asia [21].

A species of *Rosellinia*, which is the second largest genus in the Xylariaceae, was recently described to produce a novel cytochalasin, jammosporin A (**7**) [22], along with other known cytochalasans. The producer strain is an endoypte that was tentatively identified as “*R. sanctae-cruciana”* by sequencing of the ITS nrDNA (see Future Outlook Section for the validity of this approach). Jammosporin A (**7**) was found to be mildly cytotoxic against MOLT-4 cells with an IC_50_ value of 20 µg/mL. Cytochalasin C was concurrently isolated and assessed for its bioactivity showed stronger effects against MOLT-4 (IC_50_ 6 µg/mL). These activities are rather low as compared to the nanomolar inhibitory concentrations that are known from other members of the cytochalasan class.

The genus *Stromatoneurospora*, erected in 1973, was alternatively assigned to the families Hypocreales, Xylariales, and Sordariales due to inconclusive morphological characteristics. A recent finding of the type species of the genus, *S. phoenix*, finally enabled a molecular characterization using multigene phylogeny [23]. Thereby, *S. phoenix* was placed close to the coprophilous genera like *Poronia* and *Podosordaria* within the Xylariaceae. Complementarily, a screening of its secondary metabolites revealed two novel eremophilane sesquiterpenoids named phoenixilanes A (**8**) and B, which were found devoid of promising bioactivities in antimicrobial and cytotoxicity assays. Besides, several chemotaxonomically meaningful metabolites were isolated, e.g., punctaporonin B, known from *Poronia* spp., and 8,9-dehydroxylarone, reported from a *Xylaria* sp., thereby confirming the phylogenetic classification to the Xylariaceae [23]. These results highlight the significance of a combination of different techniques, like molecular phylogeny and chemotaxonomy, for taxonomic assignment within the order Xylariales.

### Cytochalasans from Xylariaceae (Fig. 3)

Several hundred representatives of the cytochalasans, fungal PKS-NRPS natural products, have been described to date [24], but still new derivatives are being continuously reported. Species of the genus *Xylaria* continue to be among the most prolific producers of these molecules [25].

**Fig. 3:**
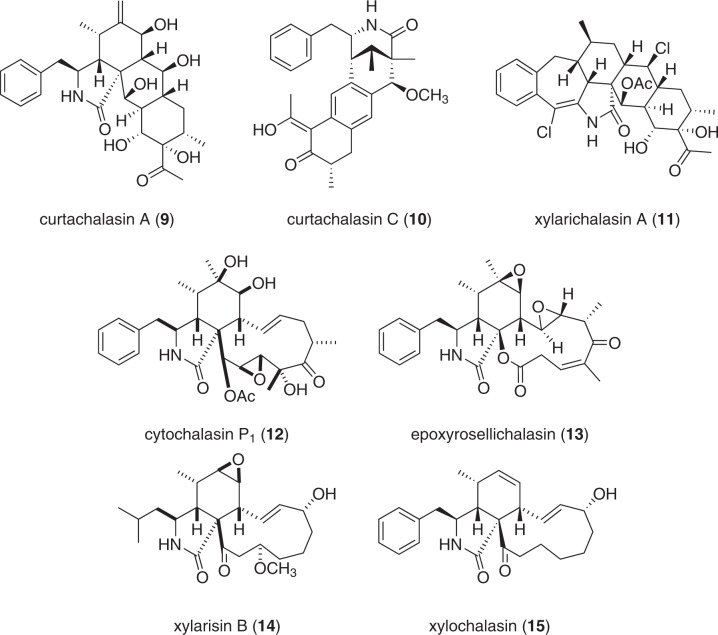
Recently reported cytochalasans from *Xylaria* spp.

A recent example is *Xylaria* cf*. curta*, which was isolated as an endophyte from potato stem tissue. Cultures of this fungus yielded numerous interesting metabolites after extensive preparative work. Among those, the curtachalasins (e.g. **9**–**10**) as well as xylarichalasin A (**11**) have to be highlighted due to their unprecedented core structures: the former harbor a tetracyclic backbone [26, 27], while in the latter, a highly complex hexacyclic structure was found [28]. Curtachalasins A (**9**) and B were found devoid of promising bioactivities in initial screenings for cytotoxicity and antimicrobial activities [27], indicating that they do not act on the actin system like other members of the class. In a subsequent publication [26], the authors assessed the ability of curtachalasins C (**10**) and E to revert fluconazole-resistance in *Candida albicans*. When **10** was given in combination with the antimycotic fluconazole, strong reversal of antifungal activity was observed. These results emphasize the vast range of bioactivity that can be exhibited by cytochalasans, once they are being subjected to a broad biological characterization. Xylarichalasin A (**11**), however, showed the strongest activity against human cancer cell lines MCF-7 (IC_50_ 6.3 µM) and SMMC-7721 (8.6 µM), besides weaker cytotoxicity against others. Notably, these activities are much weaker than those of the first reported cytochalasins, which have IC_50_ in the nanomolar range. Lately, a new derivative, curtachalasin Q, was reported from an endophytic *Xylaria* sp. isolated from roots of *Damnacanthus officinarum*, but was found devoid of cytotoxic activity [29].

A novel cytochalasin was isolated from a marine-derived *Xylaria* sp. and named cytochalasin P_1_ (**12**) [30], which is the 19,20-epoxide of the known cytochalasin P. Curiously, its cytotoxicity against human tumor cell lines was strong against SF-268 and MCF-7 (IC_50_ 1.37 and 0.71 µM, respectively), but absent against NCl-H460 or HepG2 cells (IC_50_ > 100 µM), suggesting selective cytotoxic effects.

A report of novel cytochalasans with phytotoxic activities has been published recently [31]. Besides some known cytochalasans, the novel epoxycytochalasins Z17 and -Z8, as well as epoxyrosellichalasin (**13**) have been described. Epoxyrosellichalasin (**13)** as well as the known cytochalasin K showed very strong shoot elongation inhibition against wheat (IC_50_ of 18.9 µM), which were stronger than that of the reference glyphosate (42.3 µM). The known 10-phenyl-[12]-cytochalasin Z16, cytochalasin K and E exhibited strong root elongation inhibition (IC_50_ of 17.4, 22.6, and 19.7 µM, respectively). Glyphosate, again used as a reference, showed weaker inhibition (38.1 µM). Curiously, cytochalasin K was a highly potent inhibitor of root elongation against turnip, which was ~50 times more active than glyphosate (IC_50_ 1.6 vs. 83.1 µM). Despite these interesting findings, the authors also state that the high cytotoxicity and nonavailability in ton-scales precludes cytochalasins from biotechnological applications [31]. Still, these findings highlight the potential of cytochalasans as potent agents against diverse biological targets and suggest further investigations into their mode of action.

Furthermore, the known cytotoxic compounds cytochalasin C and D were isolated from *Xylaria cubensis* and evaluated for their phytotoxic potential [32]. An assay was conducted measuring the length of wheat coleoptiles (sheath protecting the emerging shoot in monocotyledons, e.g. grasses) to assess the phytotoxicity. Both, cytochalasins C and D exhibited a stronger growth inhibition than a commercially available herbicide containing oxyfluorfen [32]. These results might be used as a starting point for further studies investigating the use of cytochalasans as crop-control agents. However, their potent cytotoxicity must also be taken into account.

Five new cytochalasans were isolated from a solid-state rice fermentation of *X. longipes* of an unreported host, along with seven known congeners. The novel compounds were found to be derivatives of known cytochalasans and showed weak cytotoxicity of IC_50_ > 40 µg/mL against human cell lines like HL-60 or A-549 [33].

From cultures of a *Xylaria* sp. isolated from the mangrove *Xylocarpus granatum*, a novel cytochalasan named xylarisin B (**14**) was isolated [34]. In assays evaluating the inhibitory effect on acetylcholinesterase (AChE)- and *α*-glucosidase, **14** was found devoid of activity.

Cultivation of a wood-decaying *Xylaria* sp. also afforded an unprecedented cytochalasin named xylochalasin (**15**) [35]. It was found to be weakly active against HeLa cells (IC_50_ 57 µg/mL), and even less active against other human cancer cell lines like HT29, HCT116, Vero, or MCF-7 (IC_50_ 90 to >100 µg/mL).

### Terpenoids and hybrid-terpenoids from Xylariaceae (Fig. 4)

A group of eremophilane sesquiterpenoids named nigriterpenes A–F was isolated from a termite-nest-derived *X. nigripes* [36]. Those compounds were assessed for their inhibitory effects on lipopolysaccharide (LPS)-induced inducible nitric oxide synthase and cyclooxygenase-2 expression in microglial BV-2 brain cells. Especially nigriterpene C (**16**) showed a strong inhibition (IC_50_ 1.8 µM) compared to the LPS vehicle control (5.7 µM), and was furthermore shown to attenuate microglial NO production in a concentration-dependant matter. Hence, the authors conclude that **16** (besides formannoxin alcohol, a known compound that was concurrently isolated) might be an interesting candidate as an anti-neuroinflammatory agent [36].

**Fig. 4:**
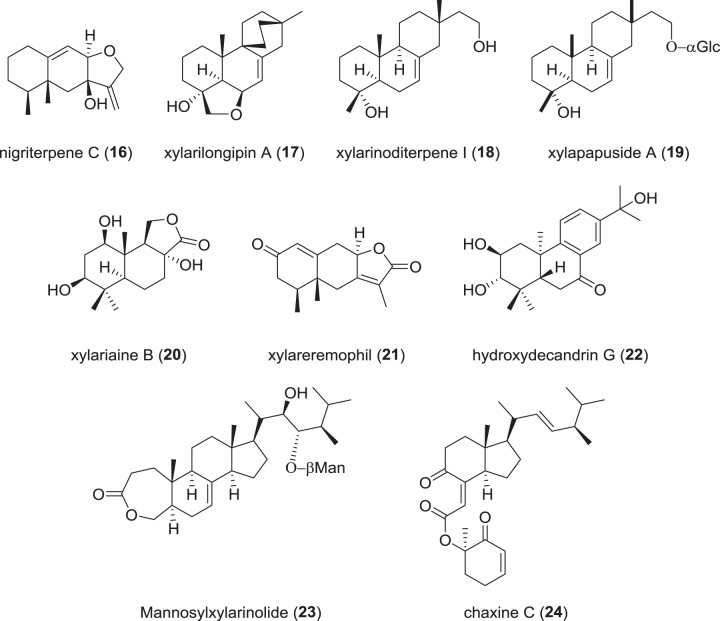
Recently reported terpenoids and terpenoid-hybrids from *Xylaria* spp.

Moreover, several *nor*-isopimarene terpenoids named xylarinoditerpenes A–R [37] and two isopimarenes designated xylarilongipins A (**17**) and B [38] were recently reported. These terpenoids were isolated from a fungicolous strain of *X. longipes*, growing on fruiting bodies of the basidiomycete * Fomitopsis betulina*. Both groups of compounds showed immunosuppressive activities against cell proliferation of concanavalin A-induced T-lymphocytes and LPS-induced B-lymphocytes. The strongest suppression was exhibited by xylarinoditerpene I (**18**) with an IC_50_ of 1 µM against induced T-lymphocytes, which indicates a strong immunosuppressive activity in comparison with the reference dexamethasone (IC_50_ 1.6 µM).

Two new isopimarene glycosides named xylapapusides A (**19**) and B were reported from cultures of an endophyte tentatively identified as *X. papulis* that had been isolated from the plant *Lepidagathis stenophylla* [39]. Compound **19** showed inhibition of NO production in LPS-induced RAW264.7 macrophages of *ca*. 34%, indicating a moderate effect as compared to the positive control, aminoguanidine (*ca*. 79% inhibition). In addition, a group of drimane sesquiterpenoids named xylariaines A–C was found in culture extracts of the same fungus [40]. These were assessed for their inhibitory effect on AChE and found to be weakly active with an enzyme inhibition of 18% exhibited by xylariaine B (**20**), as compared to the positive control tactrine (56.7%).

A number of antibacterial compounds, including a new eremophilane sesquiterpenoid named xylareremophil (**21**), were reported from a *Xylaria* sp. [41]. Xylareremophil (**21**) exhibited weak antibacterial activity (MIC: 25 µg/mL) against *Micrococcus luteus* and *Proteus vulgaris*.

A novel abietane diterpenoid named hydroxydecandrin G (**22**) from an endophytic *Xylaria* sp. [31] exhibited very strong shoot elongation inhibition against wheat (IC_50_ of 23.6 µM), which was stronger than that of the reference glyphosate (42.3 µM), suggesting potential as a natural herbicide.

In addition, a triterpene glycoside named mannosylxylarinolide (**23**) was reported from another endophytic *Xylaria* sp., but no data on biological activities were included in this publication [42].

From cultures of *X. allantoidea*, the known terpenoids demethylincisterol A3 and chaxine C were isolated [43]. Chaxine C (**24**) was moderately active against human HeLa and primate Vero cells lines with IC_50_ values of around 3 µg/mL. Chaxins, which were first reported from fruiting bodies of the edible mushroom *Agrocybe chanxingu* as active ingredients responsible for suppression of osteoclast formation [44], did not show activities against murine UAMS-32 cells. The occurrence of the rare chaxin skeleton in both, the Ascomycota and Basidiomycota is puzzling because normally these two subphyla do not share many common secondary metabolites.

### Non-ribosomal peptides from Xylariaceae (Fig. 5)

*Xylaria ellisii* was recently described as a novel species that colonizes conifers as an endophyte but forms stromata on hardwood in North America [45]. Concurrently, the fungus was studied for biologically active secondary metabolites, which revealed eight new cyclic pentapeptides designated ellisiiamides A (**25**)−H. Only ellisiiamides A–C were actually purified, while the structures of the remaining congeners were derived from LC–MS analyses. Assessment of the antimicrobial activities of ellisiiamides revealed no promising effects. Besides these pentapeptides, several known compounds like griseofulvin, cytochalasans, hirsutatin A, and piliformic acid were detected, underlining the chemical potential of this new species. The antifungal effects observed can probably be attributed to griseofulvin and the cytochalasans. In any case this study is exemplary because it provides sound taxonomic data on the endophytic *X. ellisii* and allows for manifold follow-up work on its ecology and the biological functions of its secondary metabolites. Needless to say, the molecular identification of *X. ellisii* included a multigene genealogy and detailed morphological studies, rather than just generation of rather inconclusive ITS sequences, and the fungal cultures as well as the sequence data are deposited in the public domain.

**Fig. 5:**
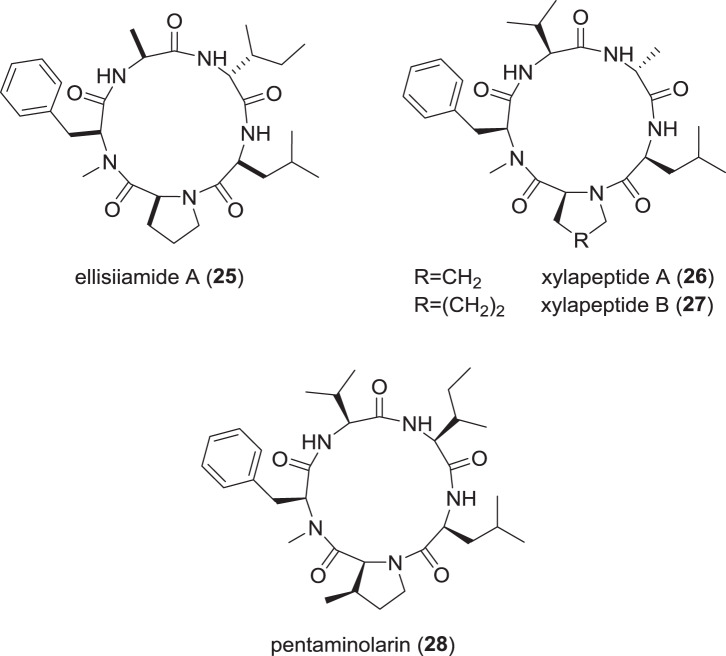
Recently reported non-ribosomal peptides from *Xylaria* spp.

Another description of cyclic pentapeptides was reported from a *Xylaria* sp. isolated from the Chinese medical plant *Sophora tinkinensis* [46]. The compounds, which were given the trivial names xylapeptide A–B (**26**–**27**), showed moderate but selective antibacterial activities. Minimum inhibitory concentrations (MIC) of **26** against *Bacillus* sp. were 12.5 µg/mL, while **27** additionally exhibited moderate antibacterial activity against various bacteria including the pathogenic *Staphylococcus aureus* (MIC 6.25–12.5 µg/mL). Furthermore, xylapeptide B (**27**) showed moderate antifungal effects against *Candida albicans* with a MIC of 12.5 µg/mL. Notably, **26** had the uncommon l-pipecolinic acid incorporated in its peptide structure, while **27** had an l-proline at this position. With this being the only difference between both compounds, contribution of this amino acid residue to antimicrobial activity is indicated. Moreover, antiviral or cytotoxic activities were evaluated but not observed.

Cultivation of a wood-decaying *Xylaria* sp. also afforded a novel cyclic pentapeptide as well as an unprecedented cytochalasin [35]. The cyclic pentapeptide pentaminolarin (**28**) showed weak inhibition against HT29 and HCT116 cell lines (IC_50_ 32 and 38 µM).

### Macrolide polyketides from Xylariaceae (Fig. 6)

Recently, a patent was published dealing with a macrolide from *X. curta* designated E1011 (**29**), which showed cytotoxic effects against several human cancer cell lines [47]. This macrolide **29** is a bisacetylated, diastereomeric derivative of the anthelmintic 14-membered macrolide clonostachydiol (**30**), which was originally isolated from the fungus *Clonostachys cylindrospora* [48], but found in a *Xylaria* sp. [49] recently. E1011 (**29**) exhibited moderate cytotoxicity against diverse human cell lines like HL-60 or A-549 with IC_50_ values of 4.9 and 25.6 µM, respectively [47]. Clonostachydiol (**30**), was also found to be weakly cytotoxic (KB: IC_50_ 39 µg/mL, NCI-H187: 17 µg/mL) [49].

**Fig. 6:**
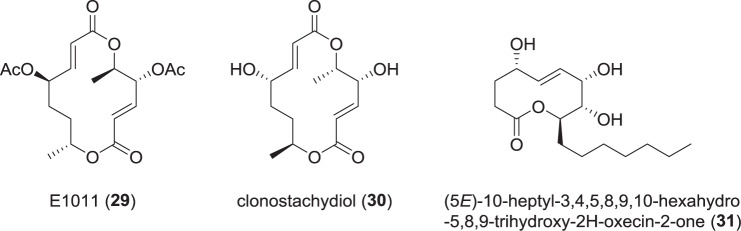
Recently reported macrolides from *Xylaria* spp.

A 10-membered macrolide was isolated from *X. feejeensis*. No name was assigned, but its systematical name is (5*E*)-10-heptyl-3,4,5,8,9,10-hexahydro-5,8,9-trihydroxy-2*H*-oxecin-2-one (**31**) [50]. It was assessed for its potential to inhibit osteoclastogenesis, as an excessive osteoclastogenesis is clinically associated with diseases like osteoporosis or rheumatoid arthritis. A strong inhibition in terms of reduced numbers as well as areas of osteoclasts was observed, which renders **31** an interesting target to study further.

### Benzenoids and lactones from Xylariaceae (Fig. 7)

A number of natural products was recently described from a termite nest-derived *X. fimbriata*. In total, seven benzoid ethers named fimbriethers A–G were isolated and characterized [51]. Assessment of their anti-inflammatory activity via a nitric oxide inhibition assay in RAW264.7 cells resulted in fimbriethers B (**32**), E, and G to exhibit moderate anti-inflammatory activity. Hence, fimbriethers may serve as agents against inflammation in mammals, given that none of them are cytotoxic against RAW264.7.

**Fig. 7:**
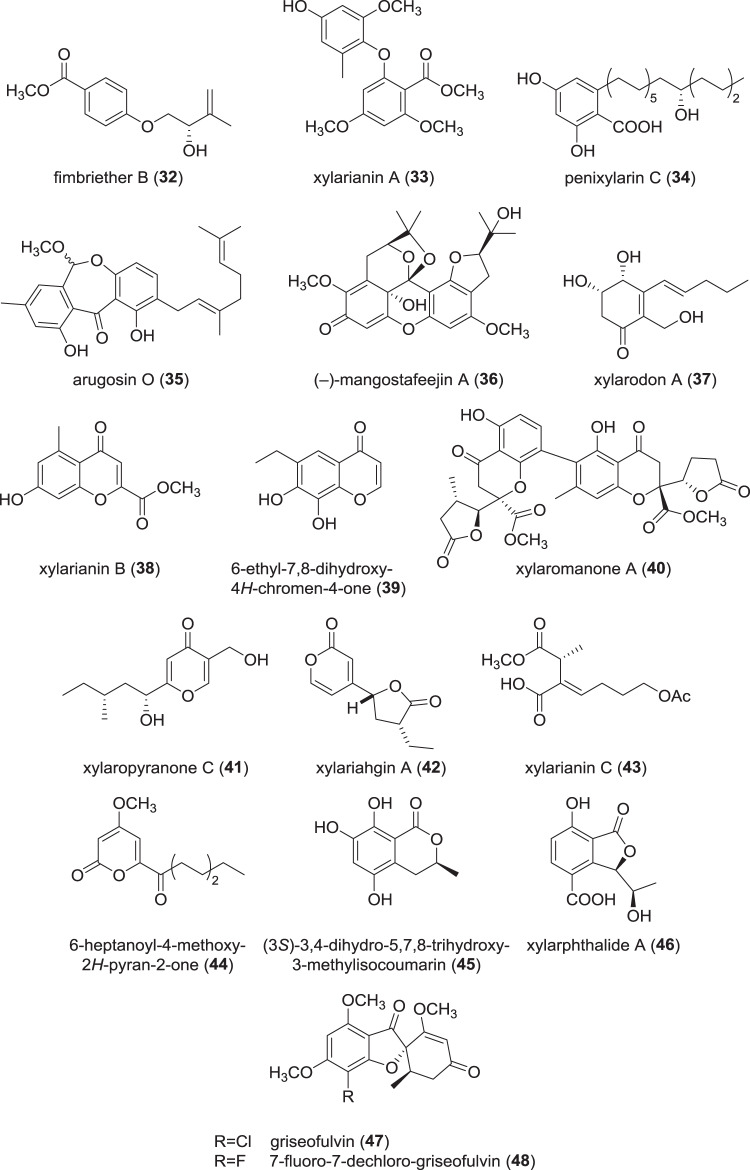
Recently reported benzenoids and lactones from *Xylaria* spp.

An investigation into the secondary metabolism of an endophytic *Xylaria* sp. from leaves of *Hevea brasiliensis* resulted in isolation of 18 compounds in total with description of the new natural products xylarianins A–D [52]. The xylarianins comprise compounds of three different classes: while xylarianin A (**33**) is an oxydibenzenoid, B (**38**) represents a chromone backbone, and C (**43**) and D are succinic acid derivatives. Along with the xylarianins, three known oxydibenzenes were isolated. All 18 compounds were evaluated for their inhibitory effect on human carboxylesterase 2 (hCE2). The oxydibenzenoids like **33** (IC_50_ 10.43 µM) were found to be moderately active in comparison to the positive control loperamide (1.31 µM), which is a selective inhibitor of hCE2 and clinically used as medication against diarrhea. Highest activity among the isolated compounds was exhibited by the known oxydibenzenoid 2-(2,4-dimethoxy-6-methylphenoxy)-4,6-dimethoxy-benzoic acid methyl ester (IC_50_ 6.69 µM).

A co-cultivation approach with *Penicillium crustosum* and a *Xylaria* sp. derived from roots of *Sonneratia caseolaris* was used to isolate four new, alkylated benzenoid natural products designated penixylarins A–D, along with two known benzenoids [53]. While the two known compounds as well as penixylarins C (**34**) and D were produced by the *Xylaria* sp. under axenic conditions, penixylarins A–B were only found in the co-cultures. Bioactivity evaluation showed that **34** in particular exhibited activity against *Mycolicibacterium phlei* with an MIC of 6.25 µg/mL. Against *Vibrio parahemolyticus*, an MIC of 12.5 µg/mL was measured. The positive control ciprofloxacin showed MIC of 1.56 and 12.5 µg/mL, respectively.

Furthermore, new dibenzoxepin derivatives named arugosins O (**35**) to Q were reported from an unidentified Xylariaceae sp. [54]. No effects were observed in assays evaluating antibacterial or cytotoxic activities. Generally, arugosins are rather widespread among fungi, given that they were reported from coprophilous, marine-derived, and endophytic genera, among others [55–57].

*Xylaria feejeensis* is biologically associated with the mangosteen fruit and was thus examined for its capability to biotransform plant metabolites to novel derivatives [58]. One of the secondary metabolites produced by said plant is the xanthone *β*-mangostin, which is associated with anti-inflammatory, antibacterial, antimalarial, and antimycobacterial activities. Cultivation of *X. feejeensis* with *β-*mangostin resulted in two novel natural products, mangostafeejin A (**36**) and B, both of which occurred as (+) and (−)-isomers. No bioassays were reported, but the results make an interesting case for investigation of the plant-fungus ecology on the secondary metabolite level.

The novel hexaketides xylarodons A (**37**) and B were isolated from an endophytic *Xylaria* sp. [59], and tested for cytotoxic effects and inhibition of tyrosine kinase but were found devoid of activity.

A number of small polyketides were characterized from an ascospore-derived strain of a *Xylaria* sp., whose stromata were collected from rotten wood [60]. Submerged cultivation of the fungus yielded two chromones, 6-ethyl-8-hydroxy-4*H*-chromen-4-one and 6-ethyl-7,8-dihydroxy-4*H*-chromen-4-one (**39**), as well as two isocoumarins, 3,4-dihydro-8-hydroxy-7-methoxy-3-methylisocoumarin and 3,4-dihydro-5,7,8-trihydroxy-3-methyl-isocoumarin (**45**). Compound **39** had weak activity against HT29 and HCT116 cells (IC_50_ 16.5 and 23.1 µg/mL), and **39** and **45** exhibited anti-inflammatory effects against LPS-stimulated RAW264.7 macrophages with IC_50_ values of 1.6 and 3.0 µg/mL, respectively. The results indicated a comparable or even slightly stronger activity than the positive control, diclofenac [60].

A number of (dimeric) chromones were reported from a *Xylaria* sp. [61] isolated from the leaves of the rubber tree *Hevea brasiliensis*. Besides known monomeric chromones, three new dimeric compounds named xylaromanones A (**40**) to C were described, which constitutes the first occurrence of such dimers from the genus *Xylaria* [61].

Two new pyranone derivatives named xylaropyranones B to C (**41**) [62] were isolated from an endophytic *Xylaria* sp. along with xylaropyranone, which is known from *X. feejeensis* [63], and annularins A and C. All except for annularin C were tested for cytotoxic and tyrosinase-inhibitory activity, but found devoid of noticeable effects. Another occurrence of pyranones was reported from an endophytic *Xylaria* sp. and named Xylariahgins A (**42**)–F [64]. An assessment of their cytotoxicity against human tumor cell lines such as HL-60, A-549, and MCF-7 was unsuccessful.

Occurrence of antibacterial metabolites was reported from *Xylaria* sp. within two publications: in the first work [65], a new pyranone, 6-heptanoyl-4-methoxy-2*H*-pyran-2-one (**44**), was reported, while the second publication gave account on a novel phthalide named xylarphthalide A (**46**) [66]. Both compounds showed weak activities against *Escherichia coli* and *Staphylococcus aureus* (**44**, MIC 50 µg/mL) or *E. coli* and *Bacillus subtilis* (**46**, MIC 12.5 µg/mL), respectively.

The antifungal agent griseofulvin (**47**), originally found in *Penicillium griseofulvum*, is well-studied and even clinically applied against dermatophytosis. However, research for more potent derivatives is ongoing using *X. cubensis* as an alternative producer. In a recent study, **47** was semisynthetically derivatized to form fluorinated analogs, as fluorine was expected to strongly alter the chemical properties when incorporated [67]. Of the eleven compounds generated, only 7-fluoro-7-dechlorogriseofulvin (**48**) showed an activity similar to **47** against the skin-infection causing *Microsporum gypseum*. While no analog was shown to be more potent than the natural product, structure–activity relationships could be deduced and a simple way of incorporating fluorine into natural products was demonstrated.

### Miscellaneous secondary metabolites from Xylariaceae (Fig. 8)

Co-cultivation is a regularly applied approach to induce activation of so-far silent gene clusters. One recent positive example of a novel backbone discovered is the meroterpenoid wheldone (**49**), which was produced (presumably) by *X. flabelliformis* when co-cultured with two different *Aspergillus* spp., respectively [68]. A moderate cytotoxicity was measured for **49** with lowest IC_50_ values of 2.4 µM against MDA-MB-435 (human melanoma cancer cell line). As the authors state that both, *X. flabelliformis* and one of the *Aspergillus* sp. were genome-sequenced, a correlation to the biosynthetic gene clusters (BGC) can be made and the actual producer of **49** proven.

**Fig. 8:**
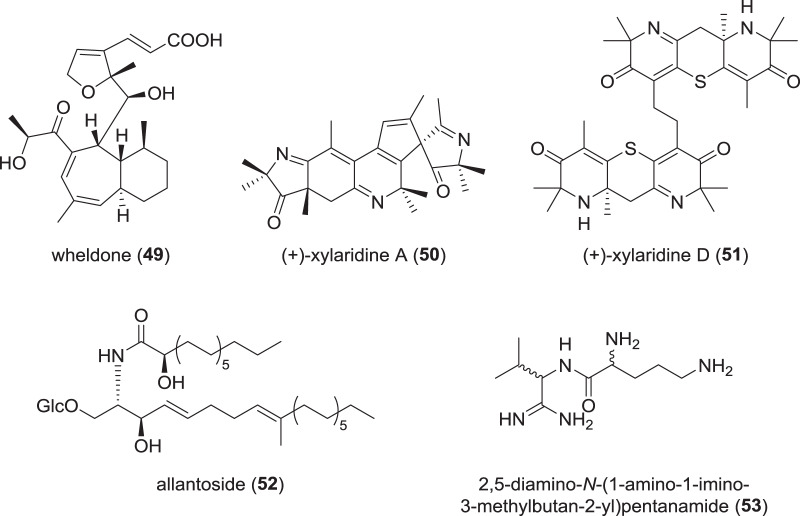
Recently reported miscellaneous secondary metabolites from *Xylaria* spp.

Another approach to attain novel natural products is heterologous gene expression. Recently, this concept has been utilized by mixing genes responsible for production of the potent statins (of which lovastatin is the most famous representative) from two producing organisms, *Aspergillus terreus* and *X. grammica*, in *Saccharomyces cerevisiae* [69]. This ultimately resulted in MS/MS predictions of two novel structures, *O*-acetylmonacolin J and “methylbutyryl DA_FR901512”. Even though neither of the new compounds were purified, application of this approach highlights the vast amount of novel chemistry that can be created using combinatorial approaches.

*X. longipes* is a fungus known for production of the antifungals xylarin and xylaramide [70, 71], as well as the succinic acid derivative piliformic acid [72]. In the past years, further progress was reported, starting with biotransformation studies of the fluoroquinolone antibiotic ciprofloxacin in 2018 [73]. The authors showed that cultures of *X. longipes* are able to derivatise ciprofloxacin, reducing its antibacterial activity by 75–88%. Before, the authors already showed similar effects when giving other fluoroquinolones to the fungus [74, 75]. These studies are very interesting examples of how fungi may be utilized for bioremediation purposes.

Furthermore, a multitude of new (thio)-alkaloids has been isolated from *X. longipes*. These compounds, named xylaridines A (**50**) to B [76], as well as thio derivatives thereof named xylaridines C–D (**51**) [77], were assigned as NRPS-PKS hybrids, which seems unlikely given the chemical structures of the compounds. Even though none of them were found to exhibit promising activities in antimicrobial or cytotoxicity assays, the biosynthesis seems to be very interesting to decipher due to the chemical complexity of the nitrogen and/or sulfur-containing structures.

In addition, derivatives of the known stromatal metabolites from *Xylaria*, xylaral and xylactam, were reported in 2017 [78]. The benzofuranon xylaral B was described along with two new isoindolinones, xylactams C–D, from fruiting bodies of *X. polymorpha*. No bioactivities were reported, but this report matches well with former examinations of stromatal secondary metabolites from *X. polymorpha* and other *Xylaria* species [79]. The xylarals are actually chemotaxonomic markers that were previously used to separate morphologically similar species like *X. mesenterica* from the hypoxyloid genus *Entonaema*.

The *X. allantoidea* culture that yielded the chaxins produced also a novel cerebroside named allantoside (**52**) [43]. However, no cytotoxicity was measured for **52**.

From stroma-derived cultures of *Xylaria cf. cubensis*, an amino-amindine, 2,5-diamino-*N*-(1-amino-1-imino-3-methylbutan-2-yl)-pentamide (**53**), was discovered along with several diketopiperazines and furanones [80]. No antimicrobial or cytotoxic activities were found for this new compound.

## Novel secondary metabolites from species of the Hypoxylaceae *sensu stricto*

The Hypoxylaceae were resurrected by Wendt et al. [81] to accommodate the genera that were formerly placed in Xylariaceae (*sensu lato*) that have a nodulisporium-like anamorph, in agreement with a multi-locus genealogy. In contrast to the Xylariaceae *sensu strictu*, many of their species accumulate large amounts of pigments and other secondary metabolites in their stromata, which are also of importance for chemotaxonomic purposes.

### Cytochalasans from Hypoxylaceae (Fig. 9)

Aside from *Xylaria* spp., species of the Hypoxylaceae like *Hypoxylon* or *Daldinia* are known for production of a multitude of cytochalasan PKS-NRPS secondary metabolites. A recent publication assessing the effects of 25 selected cytochalasans on the actin skeleton by immunofluorescence shed some light on the mode of action of these molecules [18]. Preliminary structure-activity relationships of cytochalasans were derived and it was shown that actin-disrupting effects are reversible in some cytochalasans, while being permanent in others. However, the work also described two unprecedented cytochalasans from *Hypoxylon fragiforme*, named fragiformins C and D (**54**). While fragiformin C attained incomplete actin disruption at 5 µg/mL and was partially reversible, fragiformin D (**54**) was highly active, yielding complete disruption at 1 µg/mL without reversibility [18].

**Fig. 9:**
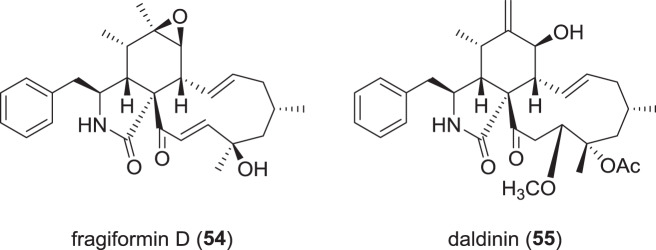
Recently reported cytochalasans from species of the Hypoxylaceae

A recent biosynthetic study investigated the function of cryptic P450 monooxygenases involved in cytochalasan biosynthesis from *H. fragiforme* as well as *Pyricularia oryzae* [82], both natural cytochalasan producers. Using combinatorial biosynthesis, six P450 genes were individually expressed in two *Δ*P450 mutants of *Magnaporthe grisea*, respectively. This led to induction of a number of unprecedented cytochalasans and indicated the functions of the tailoring P450 monooxygenases involved in cytochalasans biosynthesis in both producer organisms. Concurrently, while examining negative controls of *H. fragiforme* for the heterologous expression, a new cytochalasan was discovered and named fragiformin E, but not characterized concerning its bioactivities.

In 2019, a novel cytochalasan from fruiting bodies of *Daldinia concentrica* was isolated and named daldinin (**55**), along with two known ones [83]. Assessment of the cytotoxic activities showed that **55** exhibited weak cytotoxic activity against several cell lines like SK-LU-1 or MCF-7 with IC_50_ values of 11.4 and 13.5 µM, respectively. It has to be noted that **55** carries the same trivial name as the azaphilone pigments daldinins described from the very same fungus [84].

### Azaphilones from Hypoxylaceae (Fig. 10)

Lately, a number of fossil samples from the medieval age resembling fruiting bodies of *Hypoxylon* spp. were analyzed morphologically, microscopically, and chemotaxonomically to allow for species determination as no intact DNA was available for sequencing [85]. Combination of these three methods allowed for some of the specimens to be determined as *H. fragiforme*. Especially LC–MS analysis of the samples was noteworthy, as even after *ca*. 1000 years intact azaphilone pigments were observed, which is remarkable given the fact that these pigments are known (and even named) for spontaneous reactions with diverse nitrogen species [86].

**Fig. 10:**
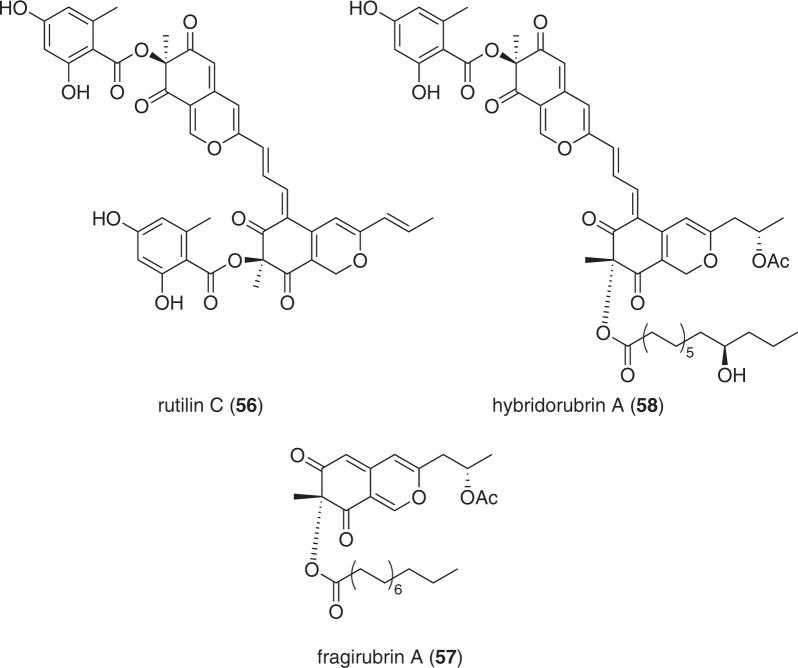
Recently reported azaphilones from species of the Hypoxylaceae

By comparing fossilized and fresh material, a number of unidentifiable peaks was found in both samples. Hence, fresh material of *H. fragiforme* was used to isolate a number of azaphilones. Besides the known mitorubrin-type pigments, two further congeners of the dimeric rutilin-type azaphilones like rutilin C (**56**), as well as the fatty acid-carrying fragirubrins A (**57**) to E were discovered [85]. Follow-up work on those compounds yielded fragirubrins F–G as well as novel heterodimers composed of mitorubrin- and fragirubrin-type azaphilones named hybridorubrins A (**58**) to D [87]. Assessment of the biofilm formation capabilities of **56**–**58** and some congeners against *Staphylococcus* *aureus* showed that the dimeric rutilins and hybridorubrins exhibit particularly strong effects, which lie in a range with the biofilm-inhibiting microporenic acid A [88]. Electronic Circular Dichroism measurements allowed for assignment of *H. fragiforme* azaphilones into three stereochemical groups. The first one contains the C-8(*R*)-configured, acyl-carrying lenormandins and fragirubrins; the second one comprises the (*S*)-configured, orsellinic acid-carrying mito-rubrins and dimeric rutilins, while the third one includes the dimeric hybridorubrins, which are composed of one (*R*)- as well as one (*S*)-configured azaphilone subunit.

Concurrently, genome analysis of *H. fragiforme* revealed two distant, cross-acting BGC to be responsible for production of different azaphilone classes and subsequent tailoring. The findings underline the potential of combining natural product chemistry and state-of-the-art genome sequencing techniques as a powerful tool.

### Terpenoids and terpenoid-hybrids from Hypoxylaceae (Fig. 11)

In 2017, a number of drimane sesquiterpenoid-isoindolinone hybrids named fendlerinines A–D (**59**) as well as drimane-phthalide compounds designated fendlerinines E–F were described from a saprotrophic fungus identified as “*Hypoxylon fendleri*” collected from Thailand [89]. It remains unclear whether the taxonomy of the strain is correct according to the current concept because this species has only been safely recorded from America (the type is from Venezuela) and there are many similar taxa in tropical Asia. However, this strain has an extraordinarily diverse secondary metabolism. Subsequent work on the fungus yielded 13 additional drimane-phthalides, namely fendlerals A (**60**)−C, fendleric acids A–C, fendlerins A–D (**61**), fendlerols A–B, and fendlerinine G [90]. Curiously, **61** even carries two drimane units. The fendlerals A (**60**) and B showed weak activities against the malaria parasite *Plasmodium falciparum* with IC_50_ of *ca*. 4 µM, as compared to the reference dihydroartemisin with and IC_50_ of *ca*. 2 nM. Furthermore, strong antibacterial effects against *Bacillus cereus* (MIC 1.56 µg/mL vs. vancomycin 1–2 µg/mL) and antifungal activity against the plant-pathogen *Colletotrichum* *capsici* (MIC of 6.25 µg/mL vs. amphotericin 3.13 µg/mL) were observed. However, the compounds also showed cytotoxicity in the rage of 5–10 µM and are obviously not selective enough to envisage any further development as antiinfective drugs.

**Fig. 11:**
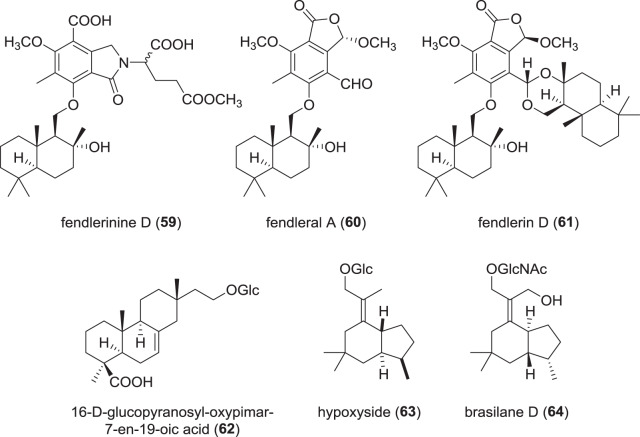
Recently reported terpenoids/hybrid-terpenoids from species of the Hypoxylaceae

A study on secondary metabolites of the related endolichenic fungus *H. fuscum* isolated from *Usnea* sp. yielded two drimane diterpenoid-glucosides 16-α-d-glucopyranosyl-(**62**) and 16-α-d-mannopyranosyl-oxyisopimar-7-en-19-oic acid [91]. Along with these compounds, a brasilane-type sesquiterpenoid-glucoside named hypoxyside (**63**) was discovered, which represents the first brasilane from the genus *Hypoxylon*. Hypoxyside (**63**) was shown to exhibit cytotoxicity against K562 cells with an IC_50_ value of 18.7 µM, which is weak compared to the reference cisplatin (3.8 µM), while for the glucoside compounds like **62** no activities were observed. Furthermore, antimicrobial assays did not show an effect of any of the isolated compounds.

A recent study of *Annulohypoxylon truncatum* revealed the presence of the known terpenoid-glucoside brasilane A, along with two unprecedented congeners named brasilanes D–E (**64**), all carrying *N-*acetylglucosamine as their glycone units [92]. Concurrent analysis of the recently published genome sequence of the fungus [93] allowed for identification of the BGC *bra* responsible for brasilane assembly. Using heterologous expression in *Aspergillus oryzae*, the functions of the respective genes in the *bra* BGC were assessed. Curiously, BraB constitutes the first example of a fungal *N*-acetylglucosamine transferase, suggesting biotechnological and/or chemical applications due to its broad substrate tolerance.

### Macrolide polyketides from Hypoxylaceae (Fig. 12)

One macrolide recently reported from a member of the Hypoxylaceae is hypoxylide (**63**), which was discovered from an endophytic *Annulohypoxylon* sp. derived from the mangrove *Rhizophora racemosa* [94]. The compound **65** features a naphthalenone moiety fused to a 10-membered lactone ring, which represents a novel backbone structure. However, no effects in assays measuring cytotoxic or antibacterial activity of **65** were observed.

**Fig. 12:**
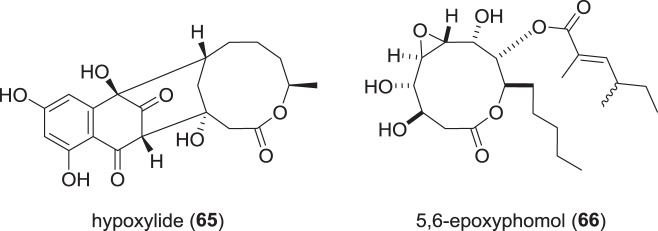
Recently reported macrolides from species of the Hypoxylaceae

Furthermore, screening of the endolichenic *H. fuscum* that also yielded hypoxyside (**63**), gave rise to another 10-membered macrolide named 5,6-epoxyphomol (**66**), besides the known phomol [91]. Both exhibited weak cytotoxicities with IC_50_ values of 15.9–32.7 µM against K562, SW480, and HepG2 cell lines, as compared to cisplatin, which showed IC_50_ values of 3.8–6.8 µM.

### Benzenoids, lactones, and other small cyclic molecules from Hypoxylaceae (Fig. 13)

Investigations of the stromata of the recently described *Annulohypoxylon viridistratum* [95] yielded three unprecedented, fully conjugated benzo[*j*]fluoranthenes named viridistratins A–C, along with the known truncatones A and C [96]. A broad-spectrum antimicrobial activity against bacteria and fungi was measured for viridistratins A and B (**67**), with the strongest activity exhibited by **67** against *Mucor hiemalis* with an MIC of 4.2 µg/mL, as compared to nystatin (66.7 µg/mL). Furthermore, **67** showed a strong cytotoxicity against the human cell lines A-431 and A-549 with IC_50_ values of 1.1 and 1.4 µM, respectively. Due to their distinctiveness, the viridistratins can serve as chemotaxonomic marker compounds for *A. viridistratum*.

**Fig. 13:**
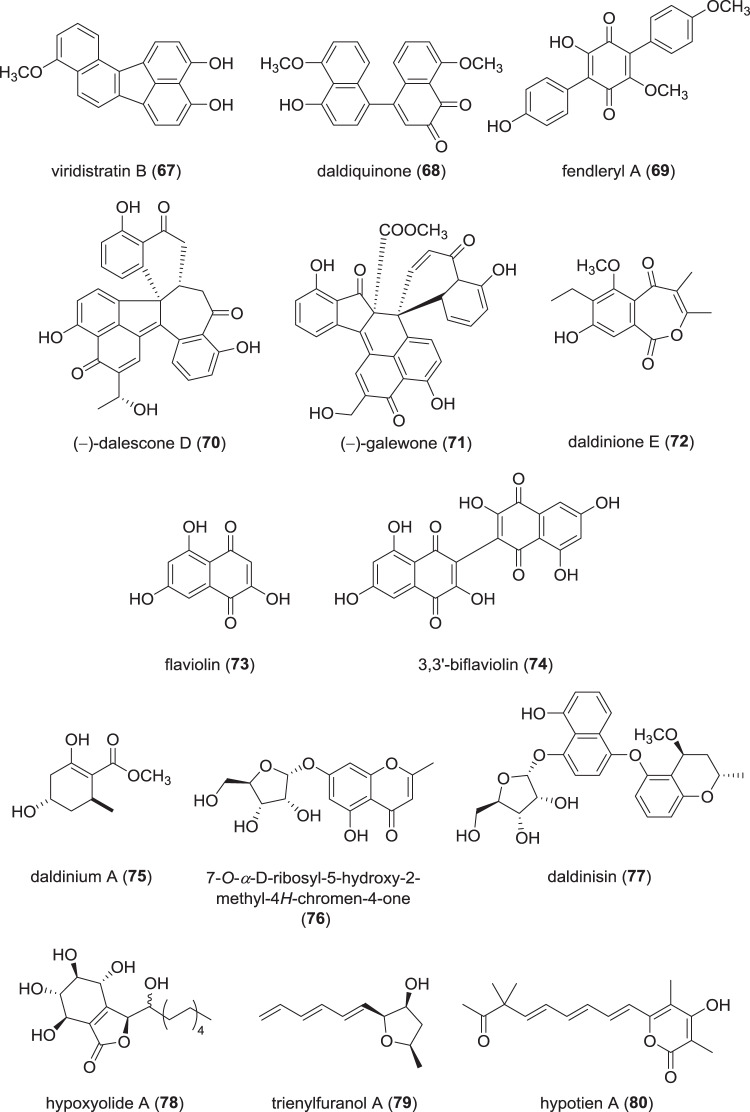
Recently reported benzenoids, lactones, and other small cyclic molecules from species of the Hypoxylaceae

Extraction of secondary metabolites from fruiting bodies of *Daldinia concentrica* led to the isolation of the novel isoindolinones daldinans B–C, the phthalides daldinolides A and B, and the binaphthalene daldiquinone [97]. Only daldiquinone (**68**) exhibited anti-angiogenesis activity measured via inhibition of human umbilical vein endothelial cells (HUVEC) growth with an IC_50_ of 7.5 µM, with cytochalasin B as positive control (IC_50_ 0.2 µM). The identification of this fungus is dubious because *D. concentrica sensu strictu* has never been safely recorded from Japan and Asia.

Within the same work reporting on a multitude of new terpenoids from a species potentially representing *H. fendleri* (see Terpenoids section), several terphenyls were reported [89]. Apart from rickenyls C–E known from *H. rickii* [98], four new terphenyls named fendleryls A (**69**) to D were isolated [89]. Fendleryls A−D exhibited no antimicrobial activity, but weak cytotoxicity against NCI-H187 and Vero cells with IC_50_ values of 48 and 49 µM, respectively. Follow-up work also afforded fendleryl E, which showed no promising bioactivities in antimicrobial or cytotoxic activity assays [90].

An approach exploiting upregulation of enzymes to obtain novel chemistry was presented in 2017 [99]. The authors cultured a *Daldinia eschscholtzii* from mantis-gut, a producer of dalesconols, and supplemented the epigenetic modifier procaine. This led to incorporation of guest intermediates into the dalesconol bioassembly lines, which resulted in isolation of the novel dalesconol derivatives (+)- and (−)-dalescones A–G. Assessment of the IL-1*β* production inhibition, whose secretion represents NLRP3 inflammasome activation, showed that especially (−)-dalescone D (**70**) exhibited a strong inhibitory effect. IC_50_ values of **70** were 3.9 µM, which is *ca*. five times more active as compared to the positive control andrographolide, which had an IC_50_ of 21.5 µM.

In a follow-up work, axenic cultivation of *D. eschscholtzii* without supplements revealed two dalesconol-related stereoisomers, (+)- and (−)-galewone (**71**), which harbor a *spiro*-connection [100]. Both isomers and the racemate were assessed for their anti-fibrotic activites; (−)-galewone (**71**) showed low IC_50_ values of 3.7 µM against fibrosis-involved CFSC-8B cells, but a comparably low activity against Lx-2 cells (IC_50_ 26.7 µM), suggested a selective effect.

Screening of cultures of *D. eschscholtzii* isolated from the mangrove *Bruguiera sexangula* yielded a number of tetralones and chromones, of which daldiniones A–E and helicasolides D–E represent unprecedented structures [101]. Curiously, daldinione E (**72**) contains an unusual 7-membered lactone ring. Bioactivity testing of those tetralones and chromones in an *α*-glucosidase inhibition assay remained fruitless.

The fungicolous *H. invadens*, which was only described in 2014, exhibits a unique lifestyle by growing on a member of its own genus, *H. fragiforme* [102]. So far, only volatile organic compounds have been described from *H. invadens* [103], but a recent investigation into its secondary metabolites revealed the known naphthoquinones flaviolin (**73**) and 3,3′-biflaviolin (**74**) to be produced in submerged cultures [104]. Only known as products of the melanin biosynthesis when the underlying pathway was actively influenced (e.g. by adding melanin biosynthesis-inhibiting fungicides [105]), **73**–**74** were found to be produced in large amounts without any intervention, but the reason for that remains dubious. Assessment of the bioactivities of **73**–**74** against microbes and mammalian cell lines did not yield interesting results.

A large number of known as well as novel benzenoids, benzopyranes, and benzopyrane-glucosides has been isolated from an endophytic *D. eschscholtzii* from *Dendrobium chrystotoxum* [106], along with one cyclohexene named daldinium A (**75**). No significant bioactivities were found in antimicrobial assays, but one glucoside, 7-*O*-*α*-d-ribosyl-5-hydroxy-2-methyl-4*H*-chromen-4-one (**76**), induced a glucose consumption rate of 17.3%, as compared to the positive controls insulin and berberine (24.8 and 24.6%, respectively). For the very same strain, cultivation in red ginseng medium led to isolation of daldinisin (**77**) [107], an unprecedented benzopyran naphthalene glucoside, and a lactone, 8-hydroxylhelicasolide. Weak anti-AChE activity was observed with inhibition of 38.8% at 50 µM for **77**, while the positive control tactrine showed 64.9% inhibition at 0.333 µM.

A screening of cultures of an endolichenic isolate of *H. fuscum* furthermore yielded several known benzopyrones and -furanones, with the new hypoxyolides A (**78**) and B belonging to the latter class [91]. Weak cytotoxicity with IC_50_ values of *ca*. 20 µM were observed against K562, SW480, and HepG2 cell lines for these compounds. Even though in this case only ITS sequence barcoding was used to assign the species, the identification seems to be reliable because *H. fuscum* is one of the species in the genus that has a specific ITS nrDNA sequence.

Several furanoids were described from a raspberry leaf-derived *H. submonticulosum* [108] (a species that was recently transferred to *Hypomontagnella* [109]). Cultivation of the fungus led to the isolation of trienylfuranol A (**79**) as well as trienylfuranones A and B. Due to instability of the three compounds, a semisynthetically obtained derivative of **79**, (1*S*,4*R*)-1-hexyl-dihydro-4-methylfuran-2(1*H*)-one, was evaluated for its fungicidal properties due to similarities of the isolated furanoids to the known antifungal agent, nystatin. An activity against *Saccharomyces cerevisiae* with 74% inhibition at 250 µg/mL was observed, which is weak in comparison to the control nystatin, which achieved complete inhibition at 10 µg/mL.

The endophytic fungus *H. investiens* was isolated from the plant *Blumea balsamifera* in a recent work [110]. Cultivation and subsequent purification yielded the novel *α*-pyrones hypotiens A (**80**) to D, but assessment of the antimicrobial and cytotoxic activities was unsuccessful.

### Miscellaneous secondary metabolites from Hypoxylaceae (Fig. 14)

From a mantis gut-derived *D. eschscholtzii* that also produced the dalescones, cultures supplemented with indole-3-carbinol yielded two (bis-)indole natural products named dalesindoloids A (**81**) to B [111]. Assessment of their cytotoxicity showed that **81** was strongly active against HL-60 cells (IC_50_ 1.0 µM), while being less active against several other cell lines. Dalesindoloid B, however, was less active in comparison, but showed a broader spectrum of cytotoxicity.

**Fig. 14:**
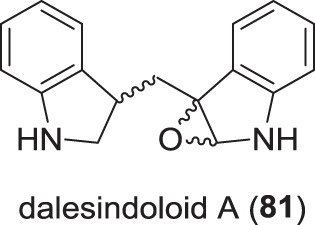
Recently reported miscellaneous secondary metabolites from species of the Hypoxylaceae

## Polythetic taxonomic, chemoecological, and phylogenomic studies on endophytic Xylariales (Fig. 15)

Recently, several papers have been published that illustrated the importance of secondary metabolites for classification or were dealing with the elucidation of the life cycle of important fungal endophytes that are members of the Xylariales. Some of these organisms have been isolated as endophytes several decades ago and were found to be producers of molecules that served as lead candidates for developmental pharmaceuticals and agrochemicals, or as promising biocontrol agents. However, their taxonomic affinities remained unclear until recently and their life cycle remained to be elucidated by means of polythetic taxonomy. A fair example for such a fungal endophyte is *H. pulicicidum*, which represents the sexual state of the endophytic *Nodulisporium* spp. that are able to produce the insecticidal and antiparasitic agent, nodulisporic acid (**82**) [112]. Cultures derived from ascospores of this fungus, which is apparently rare and forms stromata on wood, were shown to produce the compound that has been found in various endophytes that were derived from plant material collected in various tropical countries [113]. The biosynthesis of the compound has recently been elucidated [114], which offers various opportunities, e.g. to study its production *in planta* using quantitative PCR and transcriptomics and shed some light on the natural function of the molecule.

**Fig. 15:**
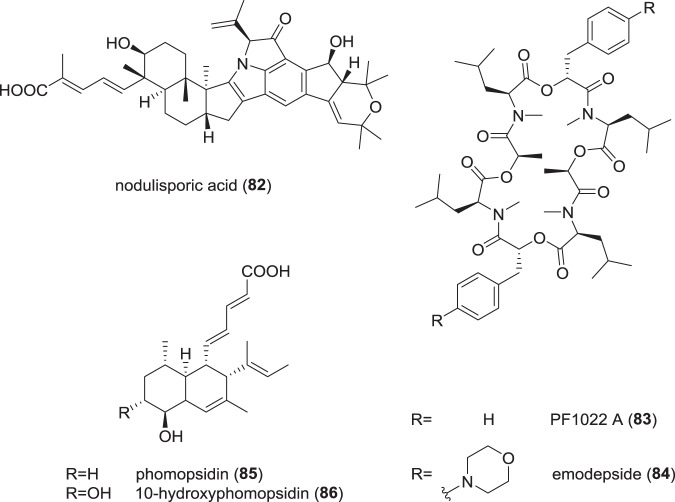
Some prominent secondary metabolites from members of Xylariales

The life cycle of the producer of the cyclodepsipeptide “PF-1022A” (**83**) [115] has also recently been elucidated in the course of the abovementioned study that also resulted in the segregation of the plant-pathogenic genus *Dematophora* from *Rosellinia* [8]. This compound is up-to-date the only one derived from an endophytic fungus that was further developed into a marketed drug. The semisynthetic emodepside (**84**), which is derived from “PF-1022A”, and can be produced in large amounts by fermentation, has been used in veterinary medicine for many years to combat worm diseases. In this case, the production is not restricted to a single species, but several strains of *Rosellinia* and the related genus *Astrocystis* were identified as producers of “PF-1022A”.

The life cycle of another important endophyte genus that contains several species which have been evaluated as biocontrol agents and so called “mycofumigants” has also recently been elucidated: the genus *Muscodor* was erected almost 20 years ago for a tropical endophyte that produces volatile antibiotics with which it kills a number of bacteria, fungal pathogens, and animal pests [116]. Over 20 species had been identified until recently and all of them were recognized on the basis of cultural morphology, volatile profiles, and molecular phylogenetic data, until recently the sexual state has been discovered from two specimens in Thailand. The ascospore-derived cultures of these fungi were able to produce volatile antibiotics, and they were found to correspond to the genus *Induratia* based on a multi-locus phylogeny. Moreover, this study also resulted in the recognition of *Muscodor/Induratia* and the related genus *Emarcea* as a unique phylogenetic lineage for which the new family Induratiaceae has been erected [117]. Interestingly, these fungi were never studied for the production of nonvolatile secondary metabolites and even the identity of the compounds that were detected by database aided GC-MS analytics often remains dubious [103, 118–120]. The Induratiaceae certainly deserve further studies of their secondary metabolome, including the identification of metabolites that show pronounced production in dual antagonist cultures.

Endophytes belonging to the genus *Hypoxylon* and its associated asexual stage *Nodulisporium* have also been repeatedly reported to produce biologically active volatiles [121, 122], even though the taxonomy of most of these strains and the identity of many of the volatile antibiotics produced by these fungi remain to be settled. Recently, an endophytic isolate that was unambiguously identified as *H. rubiginosum* was found to possess striking activities in dual culture against the Ash dieback pathogen, *Hymenoscyphus fraxineus*, and the active principles were identified after preparative isolation and classical structure elucidation (NMR spectroscopy and HR mass spectrometry) as phomopsidin derivatives [123, 124]. In a follow-up study, the production of phomopsidins (**85**–**86**) in presence of the pathogen was observed in various strains of species that are phylogenetically related to *H. rubiginosum* including the new species, *H. guilanense*, but not in some distantly related species like *H. fuscum*. Interestingly the production of the compounds, which are known to be antifungal *β*-tubulin inhibitors, was enhanced in the presence of the pathogen.

## Future outlook

The recent progress in “-OMICS” technology and bioinformatics has resulted in various options to further explore the secondary metabolism of filamentous fungi, which would have been unthinkable only 5 years ago. We wish to mention some recent highlights that concern the Xylariales and in particular the Hypoxylaceae, which have recently become a “model family” to study the correlations between phylogenomics and functional biodiversity within the fungal kingdom.

Genome sequences of their species had until recently been scarce and the few datasets that were published were mostly “shotgun” sequences based on Illumina sequencing, resulting in considerable gaps. However, the recent advent of third generation sequencing techniques like PacBio and Oxford nanopore (which still need to be complemented by “Illumina polishing” to reduce the number of sequencing errors) have substantially improved the data quality and made the generation of genome sequences much less expensive. From such high-quality genomes, it is now possible to draw much better conclusions, also because the bioinformatics tools have substantially improved in parallel. A recent study based on 13 high-quality genomes has for instance revealed for the first time that ITS sequences are present in multiple copies that may vary considerable in the same genome. These polymorphisms may in some cases explain why it is not feasible to “identify” a fungal species merely based on ITS data [125]. A larger study using these full genomes has provided the backbone for a phylogenomic study for the first time, which was based on the amino acid sequences of 4912 core genes and reflected the current accepted taxonomic concept of the family [81]. Furthermore, Percentage of Conserved Proteins analysis revealed that 70% of the proteins are conserved within the family, a value with potential application for the definition of family boundaries within the order Xylariales. Also, *Hypomontagnella spongiphila* was proposed as a new marine-derived species related to the terrestrial *Hypomontagnella monticulosa* (the type species of this recently erected genus [109]) based on in-depth genomic comparison and morphological differences of the cultures. This is the first time that high-quality genome sequence data were used to characterize species boundaries in the fungal kingdom. However, for the current topic it is more important to note that the abovementioned protein encoding genes included the complete BGCs encoding for secondary metabolites of the respective fungi, and the data can be used for synthetic biotechnology approaches. Recent studies have already made use of these data, and aside from the identification of the gene clusters by bioinformatic comparisons based on homology searches [87], it was even possible to express the BGC for cytochalasans in *M. grisea* as a heterologous host [82]. Various follow-up studies are now under way, based on these data, and exciting findings can be expected in the future from such genome mining approaches. The prerequisites to fully explore the secondary metabolome of the Hypoxylaceae and other Xylariales have therefore been created. Since the xylarialean endophyte *Pestalotiopsis fici* has already been found to contain almost 100 genes and BGC encoding for secondary metabolites using less sophisticated genome sequencing methodology [126], it can be imagined that this kind of work will result in many new discoveries of unprecedented compounds. Since the sequencing costs are steadily decreasing, the new techniques will soon be available for sequencing of large strain contingents, but skilled bioinformaticians who must continue to handle the data will never become dispensable.
